# Response shift in health-related quality of life measures in the presence of formative indicators

**DOI:** 10.1186/s12955-020-01663-y

**Published:** 2021-01-06

**Authors:** Silvia Testa, Daniela Di Cuonzo, Giuliana Ritorto, Laura Fanchini, Sara Bustreo, Patrizia Racca, Rosalba Rosato

**Affiliations:** 1 Department of Human and Social Sciences, University of Aosta Valley, Aosta, Italy; 2 Department of Psychology, University of Turin, Turin, Italy; 3 Unit of Cancer Epidemiology, “Città Della Salute E Della Scienza” Hospital, University of Turin, CPO Piemonte, Turin, Italy; 4 SSD Colorectal Cancer Unit, Dipartimento Di Oncologia, “Città Della Salute E Della Scienza Di Torino” Hospital, Turin, Italy

**Keywords:** Response shift, Formative indicators, Reflective indicators, Health-related quality of life

## Abstract

**Background:**

Response shift (RS) has been defined as a change in the meaning of an individual’s self-evaluation that needs to be accounted for when assessing longitudinal changes in health-related quality of life (HRQoL). RS detection through structural equation modeling is accomplished by adopting Oort’s procedure based on a measurement model in which the observed variables are defined as reflective indicators of the HRQoL latent variable; that is, the latent variable causes the variation in the reflective indicators. This study aims to propose a procedure that assesses RS when formative indicators are used in measuring HRQoL; in this last case, the latent variable is considered to be a function of some formative indicators. A secondary aim is to compare the new procedure with Oort’s procedure to highlight similarities and differences.

**Methods:**

The data were retrieved from a consecutive series of 258 patients newly diagnosed with colorectal cancer and undergoing chemotherapy and/or surgery. The European Organisation for Research and Treatment of Cancer Quality of Life Questionnaire (EORTC QOL-C30) was administered twice, once before and once six months after treatment. Structural equation modeling was used to evaluate RS and true change with the newly proposed method (in which fatigue and pain were defined as formative indicators) and with Oort’s procedure (in which fatigue and pain were defined as reflective indicators).

**Results:**

According to the new procedure, there was no measurement bias, and on average, patients’ quality of life improved by 3.53 points (on a scale ranging from 0 to 100) at the 6-month follow-up. With Oort’s procedure, the loading of the pain indicator was not invariant across the two time points, suggesting the presence of reprioritization, whereas the estimation of true change was very similar to the previous one: 3.87.

**Conclusions:**

RS and true change in HRQoL can be evaluated in the presence of formative indicators. Defining a measurement model by formative or reflective indicators can lead to different results.

## Introduction

The longitudinal change in patients’ perceived health-related quality of life (HRQoL) is a relevant topic in the evaluation of treatment effects and disease adaptation in individuals with chronic disease conditions [1]. Often, self-report HRQoL questionnaires are administered at two or more time points, and differences in the repeated measures are interpreted as changes in the construct of interest, assuming that the meaning of the items has not changed over this interval. However, the validity of this assumption can be undermined by the presence of the so-called response shift (RS) phenomenon [2–5].

As defined by Sprangers and Schwartz in 1999, RS refers to a change in the meaning of one's self-evaluation of a target construct due to (a) a change in the internal standards of measurement (*recalibration*); (b) a change in the importance of the component domains constituting the target construct (*reprioritization*); or (c) a redefinition of the target construct (*reconceptualization*). Although changes in the meaning of the target construct occur at the individual level, RS can also be analyzed at the group level, assuming that the majority of the cohort shows the same pattern of RS [3, 6, 7]. Moreover, RS can be evaluated at the item level—by analyzing the relationship between the patients’ rating and a narrow latent variable (typically an HRQoL domain, such as a physical, emotional or social quality of life dimension)—and at the domain level by considering the relationship between domain scores (e.g., physical, emotional, social) and the broader latent variable, HRQoL [3]. According to the model proposed in 1999 by Sprangers and Schwartz and subsequent updates [4, 5, 8], RS is triggered by a catalyst (i.e., a relevant event leading to a change in the respondent’s health status) and is related to a change in the appraisal process involved in answering HRQoL items. Thus, RS leads to a discrepancy between the observed change recorded by the patient’s self-evaluations and the true change in the construct of interest.

The structural equation modeling (SEM) approach is often used to model the relationship between the observed scores (item or domain scores) and the target construct (latent variable) [6, 9]; however, there are many studies debating the issue of measurement and reflective/formative models [10–13], and we hereby present a brief description of both types of models.

**Fig. 1:**
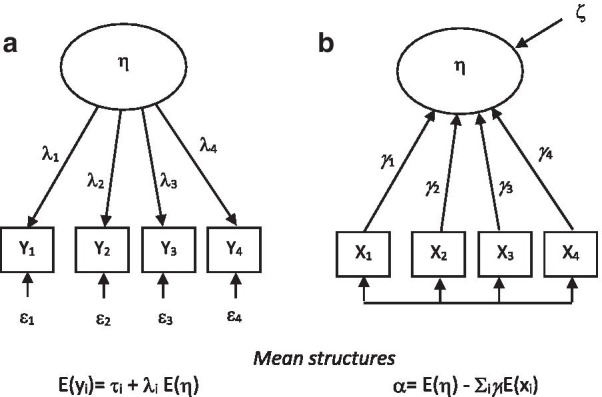
Measurement models with reflective (**a**) and formative (**b**) indicators. *Note*: **a** The latent variable η determines the score of the four items y_1_ to y_4_, all of which are measurements of the same construct. The item-level errors are depicted by ε’s, and the factor loadings are depicted by λ’s. The mean structure refers to the expected value of the observed variables. **b** The latent variable η is determined by the scores of items x_1_ to x_4_, each of which is a measurement of a different aspect of the latent construct. The weights on the construct are depicted by *γ*’s, and the error term of the construct is ζ. In the formative model, the intercept α captures the mean structure of the model. In both figures, the square boxes represent observed variables, whereas the ovals represent unobserved (latent) variables, which were estimated on the basis of the items

In a reflective model, constructs are assessed on the basis of the assumption that the underlying latent construct causes the observed variation in the observed variables. The observed measures are called reflective indicators, and they are expected to be correlated because of their dependency on the same latent constructs. For some constructs, it is more appropriate to view causality from the observed indicators to the construct. Such models are called formative, namely, it is assumed that changes in the manifest measures (formative indicators) cause changes in the underlying construct. The path diagrams of the two types of measurement models (reflective and formative) are depicted in Fig. 1.

Formative indicator models such as that shown in Fig. 1b are not identified; to solve the identification issue, the latent variable needs to affect at least two observed or latent uncorrelated variables [14]. Moreover, formative indicators do not necessarily have to be correlated, as they contribute to the definition of the construct and are not a reflection of it. For example, fatigue and pain are undoubtedly part of the multidimensional definition of HRQoL in the context of health but are not necessarily correlated (patients with a high fatigue score are not expected to have a high pain score, and patients with a low fatigue score do not necessarily have a low pain score; it can happen, but one condition does not necessitate the other). Rather, strong correlations between formative indicators, such as those described by multicollinearity in standard regression models, undermines the stability of the estimates of parameters. Last, formative indicators need to be error-free to avoid biased estimates of βs.

### Detection of RS by using SEM and reflective indicators: Oort’s procedure

From the several methodological approaches developed to address RS, Oort’s procedure [6] is one of the most attractive [3]. Oort’s procedure applies the reflective model and facilitates the detection of all forms of RS (*recalibration, reprioritization, reconceptualization*) in longitudinal studies. In Oort’s procedure, the following model links a set of observed scores y_it_ (i = 1, 2, …, S) to the target latent variable (η_t_) at time point t (t = 1,2):

1 $$\begin{array}{*{20}c} {y_{it} = \lambda_{it} \cdot \eta_{t} + \tau_{it} + \varepsilon_{it} } \\ \end{array}$$

The equation depicts the classic measurement model; λ_it_ is the loading of the indicator y_it_ on the target construct η_t_, τ_it_ is the intercept parameter, and ε_it_ is the error term, whose variance is V(ε_it_).

The three forms of RS correspond to specific changes in model parameters (λ_it_, τ_it_, V(ε_it_)) between the two time points. Specifically, when at one—and only one—of the two time points the loading is zero, a *reconceptualization* has occurred; when both loadings are not equal to zero but differ in magnitude, *reprioritization* has occurred. Changes in the internal standards of measurement are signaled by a difference in the τ parameters (uniform *recalibration*) and by a change in the error variances, V(ε_i1_) and V(ε_i2_) (nonuniform *recalibration*).

Oort’s procedure requires items/domains to be defined as reflective (effect) indicators of the target construct, i.e., the observed scores are manifestations of the latent variable [15]. However, some variables of health and quality of life are better conceived as formative (causal) indicators, as they determine the latent variable [16–18].

### Moving from reflective to formative indicators

In a measurement model with formative indicators, a set of observed scores x_i_ (i = 1, 2, …, M) at time point t determines the meaning of the target construct η_t_:

2 $$\begin{array}{*{20}c} {\eta_{t} = \alpha_{t} +_{1t} x_{1t} +_{2t} x_{2t} + \cdots + _{Mt} x_{Mt} + \varsigma_{t} } \\ \end{array}$$

where α_t_ is the model intercept, $\gamma_{it}$ is a regression coefficient indicating the contribution of each indicator to the formation of the latent variable, and ζ_t_ is the error term, with variance V(ζ_t_) (Fig. 1).

Residual variance V(ε_i_) in the reflective-type model is the variance of the indicators not explained by the common latent factor; in the formative-type model, V(ζ) is the variance of the latent variables not explained by the formative indicators.

Hence, a formative model involves intercepts (αs), weights (*γ*s) and construct residual variance (V(ζ)), which are parameters not included in a reflective model. For this reason, Oort’s procedure cannot be used and, at the best of our knowledge, a specific procedure for this type of model is not available in the literature. In the present study, we aimed to propose a procedure to detect the presence of RS when a measurement model is defined with formative indicators. The secondary objective was to compare the new procedure with Oort’s procedure to highlight similarities and differences.

## Methods

The procedure to detect RS in the presence of formative indicators was first presented and then applied in a cohort of patients with cancer to whom the European Organisation for Research and Treatment of Cancer (EORTC) quality of life questionnaire (QOL-C30). The instrument was administered at two time points, and the diagnosis was assumed as the catalyst event. Several studies have examined RS among patients with colorectal cancer (CRC) [19–22]. The EORTC QOL-C30 was chosen as the HRQoL questionnaire because previous studies in the literature investigated the nature (reflective vs formative) of its items [23, 24]. Finally, RS and true change were evaluated by the new procedure and compared to those obtained by Oort’s procedure.

### RS detection in the case of a measurement model with formative indicators: a proposal

The presence of the latent variable error term in the formative-type model is important for differentiating it from a composite measure model in which the composite variable is an exact combination of the indicators (without an error term). As stated by Bollen, “causal indicators tap a unidimensional concept (or dimension of a concept), and the latent variable that represents the concept is not completely determined by the causal indicators” (Bollen [25], p. 360, see also Bollen and Bauldry [26]). In contrast with the composite variable model, the formative-type model deals with latent variables; thus, a definition of RS similar to that proposed by Oort [6] can be applied.

The procedure we present is applicable when RS is assessed at the domain level. We will describe a situation in which two or more reflective indicators are used to overcome the identification issue, and at the end of the paragraph, we will present modifications for a situation in which two or more dependent variables are used for model identification.

The prerequisites are as follows: (a) at least two reflective indicators of the HRQoL construct that have been shown to be invariant across the two time points are available (at least metric and scalar invariant: λ_i1_ = λ_i2_ and τ_i1_ = τ_i2_ for all i), (b) the formative indicators are reliable and are not strongly correlated, and (c) changes in the mean of each formative indicator between the two time points can be assumed as true changes in the corresponding HRQoL domain (the underlying reflective indicators show at least metric and scalar invariance).

Table 1 reports the operational definitions of *reconceptualization* and *reprioritization,* which are similar to those used in Oort’s procedure. The former is a change in the set of formative indicators that measure the target construct, i.e., one or more regression coefficients that are zero at only one of the two time points; the second is a change in the magnitude of the regression coefficients from one time point to the other. In the presence of a time invariance of *γ*s, changes in the error variances imply that the variance of the latent variable that is jointly explained by the formative indicators has changed. These changes can be considered indicative of a secondary form of *reconceptualization*: at one of the two time points, specifically at that with the greater variance in error, one or more formative indicators is omitted.

**Table 1:** RS detection comparison in changes in SEM parameters between two time points

Type of change	Reflective (Oort’s procedure)	Formative (Our proposal)
Reconceptualization	Patt. (Λ_1_) ≠ Patt. (Λ_2_)	Patt. (Γ_1_) ≠ Patt. (Γ_2_)
Reprioritization	λ_i1_ ≠ λ_i2_	γ_i1_ ≠ γ_i2_
Reconceptualization (secondary)	–	V(ζ_1_) ≠ V(ζ_2_)
Recalibration (uniform)	τ_i1_ ≠ τ_i2_	–
Recalibration (nonuniform)	V(ε_i1_) ≠ V(ε_i2_)	–
True change in means	M(η_1_) ≠ M(η_2_)	f(α_1_, α_2_), where α_1_ = 0
True change in the construct variance	V(η_1_) ≠ V(η_2_)	–

Patt. means the pattern of zero and nonzero parameters in the matrix of loadings (Λ_t_) and regression coefficients (Γ_t_); V and M stand for variance and mean. For the meaning of the other symbols, see the description of Eqs. 1 and 2

Regarding the mean structure part of the model, another parameter can be tested for time invariance: the model intercept (α_t_). When the intercept at t = 1 is constrained to zero, the intercept at t = 2 informs us of the intercept change across the time points, and this change can be used to estimate the true change in the target construct. In our procedure, the model has to be estimated twice, first with mean-centered formative indicators (_f_centered_) and second with mean-centered reflective indicators (_r_centered_). In the first estimate, α_f_centered,2_ reflects the mean change, as detected by the reflective part of the model, whereas in the second estimate, α_r_centered,2_ reflects the mean change, as detected by the formative part of the model. The estimate of true change is obtained by computing the mean difference between α_f_centered,2_ and α_r_centered,2_, as detailed below.

Starting from the mean structure of the regression equation of a formative indicator model (Fig. 1), the intercept is α = E(η) − Σ_i_*γ*_i_E(x_i_), and it can be written more compactly as α = A − B, where A = E(η) and B = Σ_i_*γ*_i_E(x_i_).

When α_1_ = 0, α_2_ can be rewritten as follows:

3 $$\alpha_{2} = \left( {A_{2} - B_{2} } \right) - \left( {A_{1} - B_{1} } \right) = \left( {A_{2} - A_{1} } \right) - \left( {B_{2} - B_{1} } \right)$$

If the reflective indicators used to solve the identification issue are mean centered, the intercept is:

4 $$\begin{array}{*{20}c} {{\upalpha }_{{{\text{r-centered}},2}} = - \left( {{\text{B}}_{2} - {\text{B}}_{1} } \right) } \\ \end{array}$$

In turn, if the formative indicators are mean centered, the intercept is:

5 $$\begin{array}{*{20}c} {{\upalpha }_{{{\text{f-centered}},{ }2}} = \left( {{\text{A}}_{2} - {\text{A}}_{1} } \right)} \\ \end{array}$$

True change can be defined as the average difference between the two intercept values:

6 $$\begin{array}{*{20}c} {{\raise0.7ex\hbox{${(\alpha_{{f{\text{-centered}}, 2 }} - \alpha_{{r{\text{-centered}}, 2 }} )}$} \!\mathord{\left/ {\vphantom {{(\alpha_{{f{\text{ - centered}}, 2 }} - \alpha_{{r{\text{ - centered}}, 2 }} )} 2}}\right.\kern-\nulldelimiterspace} \!\lower0.7ex\hbox{$2$}} = {\raise0.7ex\hbox{${[(A_{2} - A_{1} ) + (B_{2} - B_{1} )]}$} \!\mathord{\left/ {\vphantom {{[(A_{2} - A_{1} ) + (B_{2} - B_{1} )]} 2}}\right.\kern-\nulldelimiterspace} \!\lower0.7ex\hbox{$2$}}} \\ \end{array}$$

As can be seen from Eq. 3, if data are not mean centered, the intercept ${\upalpha }_{2}$ does not capture the joint contribution of the variation in the reflective indicators, which is reflected in the $\left( {A_{2} - A_{1} } \right)$ term, and of the variation in the formative indicators, which is reflected in the $\left( {B_{2} - B_{1} } \right)$ term. Their joint contribution is taken into account by computing the mean of the intercepts estimated after centering the data once with respect to the formative indicators and once with respect to the reflective indicators (Eq. 6).

Let us now consider how to adapt the procedure to the case in which two or more dependent variables (instead of two or more reflective indicators) are used to fix the identification problem. The procedure is the same as before, except regarding the first prerequisite and the way in which true change is evaluated. The first prerequisite states that the two or more dependent variables are mean centered, while the assessment of true change requires a single model estimation, and the true change is directly estimated by the α_2_ parameter.

### Study cohort and data-collection procedures

The study was a prospective study aimed at evaluating changes in quality of life in CRC patients between the time of diagnosis and the six-month follow-up. Two hundred fifty-eight participants were enrolled at the cancer care unit at the Città della Salute e della Scienza Hospital in Turin between October 2014 and October 2016. The inclusion criteria were a new diagnosis of CRC and an age older than 18 years. Patients with previous neoplasms, cognitive disorders (clinical judgment) or insufficient understanding of the Italian language were excluded. In this prospective study, patients were enrolled at diagnosis during the first multidisciplinary visit to determine the appropriate chemotherapy treatment and were re-evaluated at the six-month follow-up visit (between April 2015 and April 2017). At baseline, the respondents completed forms pertaining to demographic information and their self-reported health status and mood disorder questionnaires; the self-reported questionnaires were readministered at the follow-up visit. A member of the research team was available to help fill out the questionnaire. The study was conducted in strict accordance with the ethical guidelines of the Declaration of Helsinki and was approved by the Human Research Ethics Committee at the Città della Salute e della Scienza Hospital (registration number 0077310). All participants were informed about the study and consented to participate. They were assured that participation was voluntary. The participants were also informed that their refusal to participate would not affect their care.

### Measures

The QLQ-C30 is composed of 30 items that measure six functional dimensions (emotional, physical, global health, cognitive, role and social) and eight symptoms (appetite loss, constipation, fatigue, nausea/vomiting, pain, diarrhea, dyspnea and insomnia); only one item is related to financial problems. The dimension scores range from 0 to 100. For the functional dimensions, a higher score represents a better quality of life, while for the symptoms scales, lower values indicate a better quality of life [27].

### Model specification and data analyses

#### RS detection and the assessment of true change with the formative indicator model

**Fig. 2:**
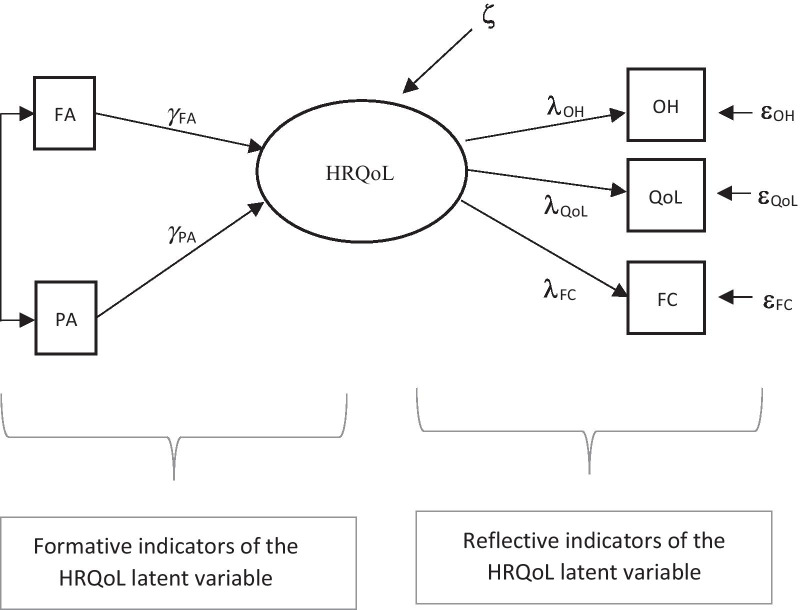
Formative model for HRQoL latent variable based on the domain indicators of the EORTC QOL-C30 questionnaire. *Note*: OH, overall health; QoL, overall quality of life; FC, functioning scales; PA, pain; FA, fatigue; γ referring to the contribution (weights) of formative indicators (FA and PA) to the latent variable (HRQOL); ζ represents the error or residual of the latent variable (HRQOL); ε refers to the error term for the reflective indicators (OH, QoL, FC); λ indicates the loading of the reflective indicators (OH, QoL, FC) on the latent variable

According to Boehmer and Luszczynska, physical symptoms were considered formative indicators [16]. More specifically, among the physical symptoms included in the EORTC QOL-C30 questionnaire, only fatigue (FA) and pain (PA) were used; they are also used as reflective indicators in the HRQoL questionnaire [28, 29], thus allowing us to compare the two alternative specifications. Three reflective indicators were considered to resolve the issue of model identification: the two items of the global health subscale, overall health (Q29, OH) and quality of life (Q30, QoL), and a global measure, which was the average of the remaining five functional subscale scores (FC). Hence, two formative and three reflective indicators were specified in the formative indicator model (Fig. 2).

Before going into the details of the procedure, it is worth noting that the model specification depicted in Fig. 2 is the same as that of a multiple indicator multiple cause (MIMIC) model with the mean structure part included, although the parameters are interpreted differently [11]. A latent variable exists that receives some arrows and emits some others; however, while the emitted arrows have the same interpretation as reflective indicators that is they are observed expressions of the latent variable (multiple indicators), the receiving arrows are interpreted as formative indicators in a formative model (contributing to define the latent variable meaning) and as explanatory variables (multiple causes) in a MIMIC model. Formative indicators are conceptually different from explanatory variables because they are viewed as defining characteristics of the construct and eliminating one of them may alter the conceptual domain of the construct.

RS detection and the assessment of true change were performed in four steps.

*Step 1* The measurement invariance of the reflective indicators of HRQoL (OH, QoL and FC) that were included to resolve the identification issue was assessed. A baseline model with no equality constraints among the parameters between the two time points and a model with equality constraints on loadings, intercepts and error variances were estimated and compared. The fit of the baseline model was evaluated according to the following criteria: root mean square error of approximation (RMSEA) ≤ 0.08 and comparative fit index (CFI)  ≥ 0.95 [30, 31]. The chi-square difference test (Δχ^2^) was used to compare the fit of the two models.

*Step 2* The measurement invariance of the reflective indicators of the fatigue and pain constructs was assessed, which was averaged to create the two formative indicators (PA and FA). As in Step 1, the baseline model was compared to the constrained model. The same statistical analysis as that used in Step 1 was used to assess and compare the fit of the models.

*Step 3* For the assessment of RS, a baseline model in which the reflective part of the model was specified, as a result of Step 1, was established, and parameters of the formative part of the model were freely estimated (except for the mean of the latent variable at t = 1, which was set to zero). Both intercepts were set to zero, whereas here and in the following models, the covariances between formative indicators were freely estimated. The baseline model was compared to a constrained model, in which the regression coefficients of the formative indicators and the variance of the latent variable were set to be equal between the two time points. The same statistical analysis as that used in Step 1 was used to assess and compare the fit of the models. A statistically significant value for Δχ^2^ was considered to be indicative of RS, and modification indices (MI) were used to identify the source of the lack of invariance.

*Step 4* Estimation of the final model, with relaxed untenable constraints, and an evaluation of the true change.

**Fig. 3:**
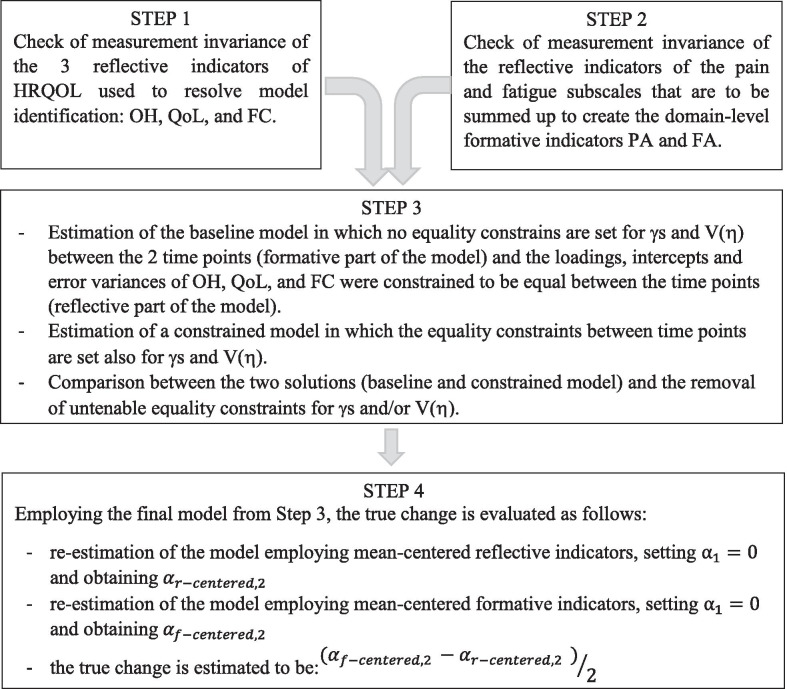
Flow chart of the procedure proposed for RS detection and the assessment of true change with the formative indicator model

The four steps are summarized in Fig. 3.

#### RS detection and assessment of true change with the reflective indicator model (Oort’s procedure)

The five QLQ-C30 indicators previously described (FA, PA, OH, QoL and FC) were defined as reflective indicators of the latent HRQoL construct. For RS detection and the evaluation of true change, the original Oort’s procedure was used [6].

SEM analyses were performed under MPLUS v7.3. The robust maximum likelihood (MLR) method was used for the estimation because the normality assumption was violated.

## Results

A cohort of 258 CRC patients completed the QLQ-C30 after diagnosis and 6 months later. The missing data rate was not greater than 2% for any of the items. For the items included in the analysis, there were no missing data.

Two-thirds of the participants were diagnosed with colon cancer (N = 162, 62.8%), the remaining participants had rectal cancer, approximately 80% of the participants underwent surgery (N = 213, 82.6%), and 51.4% of the participants were treated with chemotherapy. Almost half of the patients were men (N = 55.4%), and only 34.5% had a high school degree. The participants’ ages ranged from 30 to 89, with a mean of 67.5 (standard deviation 10.5).

Table 2 shows the baseline and follow-up descriptive statistics of the five QLQ-C30 indicators included in the SEM models.

**Table 2:** Descriptive value of the QLQ-C30 indicators included in SEM models at baseline (T1) and at the six-month follow-up (T2)

QLQ-C30 indicators included in SEM models	M	SD	Skew	Kurt
	T_1_; T_2_	T_1_; T_2_	T_1_; T_2_	T_1_; T_2_
Overall health (Q29, OH)	66.7; 70.7	23.3; 22.2	− 0.47; − .66	− .11; .13
Overall QoL (Q30, QoL)	67.9; 73.0	24.2; 22.3	− .54; − .58	− .43; − .41
Functioning scales^a^ (FC)	81.9; 85.8	14.7; 14.9	− 1.50; − 1.58	2.59; 2.65
Fatigue (FA)	30.7; 25.8	22.8; 24.7	.74; 1.03	.37; .69
Pain (PA)	17.6; 11.7	22.4; 20.2	1.30; 2.15	1.02; 4.73

^a^Average score of the five functioning sub-scales (physical, role, cognitive, social, emotional)

T1 = baseline; T2 = six-month follow-up

### RS detection and the assessment of the true change with the formative indicator model

*Step 1* As shown at the top of Table 3, the baseline model for the reflective indicators of HRQoL (OH, QoL and FC) fit the data perfectly since every reflective model with three indicators is exactly identified. With regard to the constrained model, the fit was good (RMSEA = 0.04, CFI = 0.99), and the magnitude of worsening with respect to the baseline model was not statistically significant (Δχ^2^ (8) = 13.13, p = 0.108).

**Table 3:** Fit statistics for the formative indicator models

Model	χ^2^	df	*p*-value	RMSEA	CFI	Δχ^2^	Δdf	*p*-value
Step 1: invariance of reflective indicators (OH, QoL, FC) used for identification Purpose								
Baseline	2.59	3	0.458	0	1	–	–	–
Constrained model^a^	16.06	11	0.139	0.04	0.99	13.13	8	0.108
Step 2: invariance of items used to create the two domain-level formative indicators (PA, FA)								
Baseline	30.30	24	0.175	0.03	0.99	–	–	–
Constrained model^b^	74.14	35	< 0.001	0.07	0.97	44.56	11	< 0.001
Constrained model^c^	43.26	33	0.109	0.03	0.99	13.01	9	0.162
Step 3: RS detection—invariance in the formative indicator model								
Baseline	51.59	30	0.01	0.05	0.98	–	–	–
Constrained model^d^	54.80	33	0.01	0.05	0.98	3.59	3	0.310

Step 1–step 3 are illustrated in Fig. 3

OH, overall health; QoL, overall quality of life; FC, functioning scales; PA, pain; FA, fatigue; RMSEA, root mean square approximation; CFI, comparative fit index

^a^Loadings, intercepts and residual variances of the three indicators, OH, QoL and FC, are constrained to be equal across the two time points; the mean of the latent variable is set to zero at t = 1 and freely estimated at t = 2

^b^Loadings, intercepts and residual variances of the five items measuring the latent variables PA and FA are constrained to be equal across the two time points; the means of the latent variables are set to zero at t = 1 and freely estimated at t = 2

^c^After removing two error variance equality constraints

^d^PA and FA regression coefficients and the unexplained variance of the latent variable is constrained to be equal across the two time points and the model intercept is set to zero at t = 1 and freely estimated at t = 2

*Step 2* The baseline model for the formative indicators of HRQoL, i.e., those relating to the five items measuring pain and fatigue, fit the data well (RMSEA = 0.03, CFI = 0.99). The magnitude of worsening in fit of the constrained model (with equality constraints on loadings, intercepts and uniqueness between the two time points) was statistically significant (Δχ^2^ (11) = 44.56, p < 0.001) but was not statistically significant after the equality constraints on two error variances were removed (Δχ^2^ (9) = 13.01, p = 0.162).

**Fig. 4:**
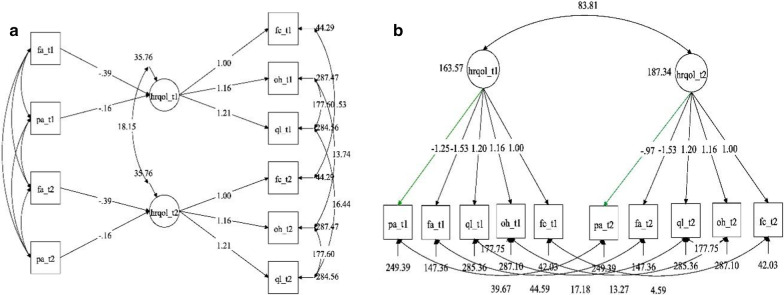
Final fully invariant formative model (**a**) and final partially invariant Oort’s procedure model (**b**). Nonstandardized estimates. *Note*: oh = overall health item; ql = overall quality of life item; fc = mean score for the functioning scales; fa = fatigue subscale score; pa = pain subscale score. The green arrows indicate noninvariant parameters; suffix t1 and t2 refer to the two time points

*Step 3* The RS baseline model performed well (RMSEA = 0.05, CFI = 0.98). When the invariance constraints were imposed on the regression coefficients and on the construct residual variance, the fit of the model was not statistically worse than that of the baseline model (Δχ^2^ (3) = 3.59, p = 0.310), leading to the final formative model in Fig. 4a.

*Step 4* As the invariance condition was fulfilled in Step 3, no RS was present according to the formative indicator model. To obtain evidence about true change in the HRQoL scores, the model intercepts were considered. In the model in which the formative indicators were mean centered, the intercept at t = 2 (α_f_centered,2_) was 4.00 (p < 0.001), and in the model in which the reflective indicators were mean centered, the intercept at t = 2 (α_r_centered,2_) was − 3.05 (p < 0.001). The estimated true change was:

(α_f_centered,2_ − α_r_centered,2_)/2 = (4.00 + 3.05)/2 = 3.53.

### *RS detection and assessment of true change with the reflective indicator model (*Oort’s procedure*)*

In the Oort’s procedure baseline model, we imposed the same invariance constraints relating to the three indicators OH, QoL and FC, as we did in Step 3 of our procedure, to guarantee the comparability of the results between the two RS procedures. As shown in Table 4, the baseline model fit well with the data (RMSEA = 0.04, CFI = 0.99), but when invariance constraints were imposed on the factor loadings, intercepts and residual variances relative to the FA and PA indicators, the magnitude of worsening in fit was quite significant (Δχ^2^ (7) = 14.45, p = 0.044).

**Table 4:** Fit statistics for the reflective indicator models

Model	χ^2^	df	*p*-value	RMSEA	CFI	Δχ^2^	Δdf	*p*-value
Baseline	51.54	34	0.027	0.04	0.99	–	–	–
Constrained model^a^	66.48	41	0.007	0.05	0.98	14.45	7	0.044
Constrained model^b^	61.05	40	0.018	0.04	0.98	9.45	6	0.150

df, degree of freedom; RMSEA, root mean square approximation; CFI, comparative fit index

^a^Loadings, intercepts and residual variances of the five indicators, OH, QoL and FC, PA and FA, are constrained to be equal across the two time points; the mean of the latent variable is set to zero at t = 1 and freely estimated at t = 2

^b^After removing the equality constraints between the PA loadings

Based on MI values and theoretical considerations, the equality constraint on the PA loading was relaxed (Fig. 4b), obtaining a model fit that was not significantly different from that of the baseline: Δχ^2^ (6) = 9.45, p = 0.150. The loading of the pain indicator was -1.25 at t = 1 and −0.97 at t = 2, meaning that pain was a better indicator of HRQoL at t = 1. In terms of RS detection, this result suggests that a reprioritization has occurred: at t = 1 pain seems to be a more important component of HRQoL than during follow-up. The estimated true change with respect to the last model was 3.87.

## Discussion

In this study, a procedure was proposed to detect RS when the indicators of the target construct are formative. According to this proposal, measurement models with formative indicators allow *reconceptualization* and r*eprioritization* forms of RS to be detected, whereas *recalibration* is not an issue; the intercepts and residual variance of the formative indicators are not model parameters. The true change in HRQoL can be estimated on the basis of two estimations of the model intercept, once by mean centering the variables used to solve the identification issue and once by mean centering the formative indicators. A measurement model with only formative indicators is not identified; to solve the problem, the target latent variable should emit at least two paths. These two paths can be related to two formative indicators or two other latent or observed uncorrelated variables that depend on the target latent variable. According to Jarvis et al. (2003), whenever possible, it is preferable to use two or more reflective indicators as “(a) the formative construct is identified on its own and can go anywhere in the model, (b) one can include it in a confirmatory factor model and evaluate its discriminant validity and measurement properties, and (c) the measurement parameters should be more stable and less sensitive to changes in the structural relationships emanating from the formative construct” (p. 213).

Our procedure can be applied when the formative indicators are at the domain level and each domain indicator is measured by two or more time-invariant reflective indicators. This is because the mean scores of formative indicators contribute directly to the formation of the latent variable mean score and to the evaluation of true change across time points. Hence, they should not show uniform recalibration.

The procedural steps proposed for the assessment of *reconceptualization* and *reprioritization* are the same as those used by Diamantopoulos and Papadopoulos [16] for assessing the cross-nation invariance of formative indicators, whereas the last step regarding the mean structure part of the model is a completely novel proposal but essential when assessing true change in a construct of interest.

In the empirical example, the newly proposed procedure was applied to a model with three reflective indicators and two formative indicators of the latent variable, HRQoL, and this procedure was compared to the traditional procedure applied to the model, in which all five indicators of HRQoL were modeled as reflective indicators. As a result, the estimated true change values for the proposed and traditional models were quite similar, as they were 3.53 and 3.87, respectively, but in terms of a lack of measurement invariance across two measurement occasions, the results differed. Defining fatigue and pain as formative indicators led to parameter estimates that were time invariant, whereas when the two indicators were defined as reflective, the loading of pain was not invariant between the two time points, and this lack of invariance could be interpreted as RS. In terms of these findings, it suggests the presence of reprioritization: pain was less important at the follow-up than at baseline. Thus, defining the indicators as formative or reflective can lead to different results in terms of the measurement invariance across time points and thus in terms of RS assessment.

It is critical that the appropriate model specification (reflective vs formative) is selected because measurement mis-specification leads to biased parameter estimates [15]. Whether an indicator is reflective or formative can be evaluated by means of empirical tests [32] or established on the basis of theoretical grounds, as the nature of the indicators depends on the definition of HRQoL that the researcher is adopting [33].

Regarding the limitations of the study, the new procedure was tested in only a single cohort and a single HRQoL questionnaire; it is not possible to generalize our findings of a few differences between the two RS detection approaches, as the results may be specific to the cohort and measures included in this study. The inclusion of additional external criteria for assessing RS would have helped us ascertain which of the two models was more specific since the fit indices of the two baseline models were quite similar. Moreover, future simulation studies are required to determine the validity of the proposed method.

In this study, RS was assessed from a measurement perspective, and future work is needed to integrate the presence of formative indicators within SEM models that investigate RS from a conceptual perspective [34, 35]. The HRQoL latent construct and its indicators (reflective and formative) can be analyzed in a network of relationships with other constructs or sociodemographic variables to detect possible bias both in the explanation and in the measurement of HRQoL.

## Conclusion

An assumption in the use of HRQoL questionnaires is that they are invariant over time, i.e., they are able to capture the real change in the interviewees even in presence of the psychological adjustments that can occur when people are facing novel situations. This means that responses to HRQoL measures over time may vary not only because health or quality of life has changed but also because people may have changed their perception of what health or quality of life means to them (RS). In assessing RS and true change by means of the SEM approach, some indicators are better conceived as reflective, whereas others as better conceived as formative. The current literature does not provide methodological tools for assessing true changes in HRQoL when the measurement model includes formative indicators. The procedure proposed in this study aims to fill this gap.

## Data Availability

Not applicable.
